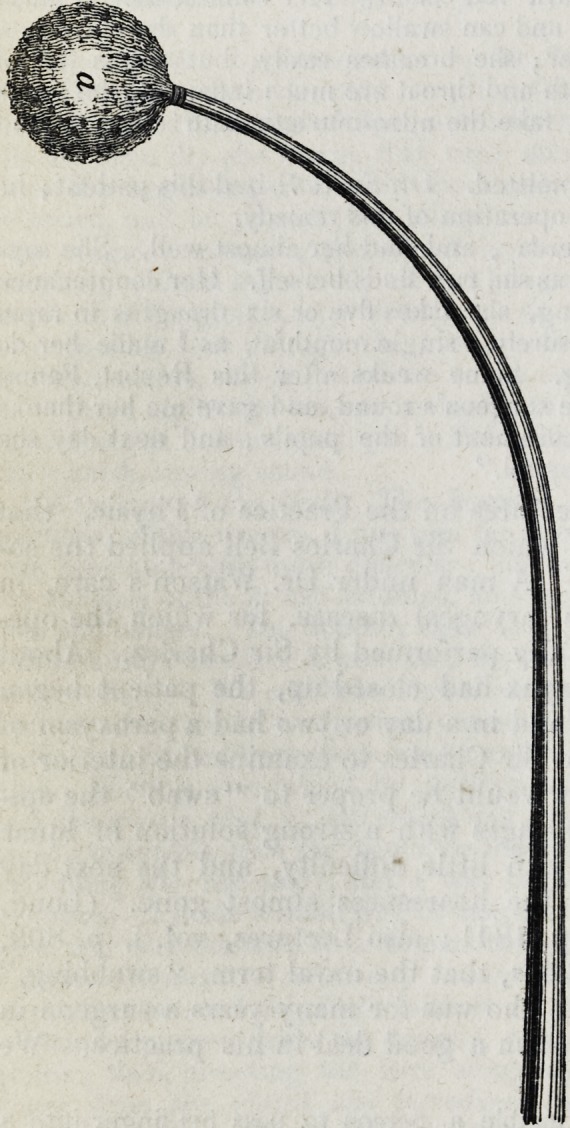# A Treatise on Diseases of the Air-Passages: Comprising an Inquiry into the History, Pathology, Causes, and Treatment of Those Affections of the Throat Called Bronchitis, Chronic Laryngitis, Clergyman's Sore Throat, &c.

**Published:** 1847-10

**Authors:** 


*A Treatise on Diseases of the Air-passages: comprising an Inquiry into the History, Pathology, Causes, and Treatment of those Affections of the Throat called Bronchitis, Chronic Laryngitis, Clergyman's Sore Throat, &c. &c.* By Horace Green, a.m., m.d., formerly President and Professor of the Theory and Practice of Medicine in the Castleton Medical College, Vice-President of the New York Medical and Surgical Society, and Honorary Member of the Philadelphia Medical Society, &c. &c.—*New York and London*, 1846. 8vo, pp. 256.

Dr. Green's work is a monograph on diseases of the follicles which stud the mucous membrane of the fauces, pharynx, and larynx, and is a practical work of very considerable merit. Its object is to point out the great value of nitrate of silver as a local application to the cavities of the larynx and pharynx, in cases of chronic laryngitis, bronchitis, and allied affections, many of which are so nearly similar in their symptoms to more serious pulmonary disease as to be mistaken for phthisis pulmonalis, or so urgently complicate the latter affection, as to render their treatment of importance, even in the alleviation of the sufferings to which the moribund of phthisis are so often exposed.

Dr. Green commences his essay with the anatomy of the fauces, pharynx, and larynx. The mucous membrane lining these passages to the alimentary and respiratory organs is studded more or less freely with glandular follicles or muciparous glands, which are analogous to the glandules of other mucous surfaces. From Dr. Green we learn, that in the mucous membrane, which unites the base of the tongue with the epiglottis, are clustered together several follicles, having their excretory ducts opening into a common dilated orifice, the *foramen cæcum,* situated at the back of the tongue. Placed near this opening, are the *lenticular papillæ* of the tongue; they consist of twelve or fifteen large mucous follicles, of a conoid shape, disposed in two lines, which converge to an angle just before the foramen cæcum. The *amygdalæ* or *tonsils* seem to be composed entirely of an aggregated mass of follicles, enveloped in folds of the mucous membrane. On the internal and convex surface of these bodies are seen a large number of deep and irregular sulci or depressions. The walls of these cavities are lined by mucous membrane, whose surface presents numerous small apertures which lead into follicles or cells, that secrete the mucous and viscid fluid with which the cavities are generally filled. The *glands of the pharynx* are large and abundant; they are of an ovoid form, and are situated beneath the mucous membrane, but are not imbedded in the submucous tissue, as are those of the œsophagus and trachea. These glands are particularly numerous around the posterior nares, and under the cervical portion of the pharyngeal membrane; two of them, more complicated in their structure, being lobulated, and of larger size than the rest, are situated at the margin of the opening of the Eustachian tube. The *follicles of the uvula* are also large, and they are particularly numerous around the inferior extremity of this organ. The *œsophageal glands* are like those of Brunner, and are imbedded in the submucous tissue of the œsophagus; they are composed of small lobulated bodies or cells, several of them having their excretory ducts united in one common tube, which opens upon the surface of the œsophageal membrane. In the *larynx,* the mucous follicles are very numerous in that part of the lining membrane which occupies the upper part of this organ. On its surface may be seen the openings of some sixty or seventy excretory tubes, which pass into follicular cells situated in the submucous tissue. Placed in the substance of the epiglottis are numerous other glandulæ which have their openings on the laryngeal surface of this structure; one of these, consisting of several granules, is imbedded in a mass of fat which is situate between the epiglottis and the os hyoides; ducts pass backwards from it through foramina in the epiglottis, to open upon the posterior or laryngeal surface of this cartilage; it is named the *epiglottotic gland.* Other glands are placed in the thickness of the superior vocal cords within the ventricles of the larynx, and in the folds of the mucous membrane in front of the arytenoid cartilage. The secretion from the laryngeal follicles is intended for the lubrication of the vocal ligaments, and it is directed upon them by small valvular folds of the mucous membrane, which are arranged in such a manner as to effect this object. The *follicles of the trachea* are still more numerous than those of the larynx; they are small, flattened, ovoid cells, situated between the fibrous and muscular layers of the membranous portion of the trachea, and in some places, beneath the muscular fibres; so that their excretory ducts have to penetrate not only the muscular layer but the mucous membrane, in order to open upon its interior surface. In their normal condition, the fluid secreted by the mucous follicles of the air-passages is bland and transparent, and not abundant in quantity; but disease, as we shall find, greatly increases and vitiates their secretion.

Previously to discussing the pathology of these structures, Dr. Green observes that affections of the throat are ordinarily yet incorrectly arranged with those of the œsophagus:

"Pathologically considered, the relation which exists between the fauces, tonsils, and pharynx, on the one hand, and the respiratory tubes, on the other, is much more intimate and important than the connexion which exists between the throat and the œsophagus. In almost all the inflammatory affections of the air-passages, whether primary or consecutive, the diseased action has its origin in the fauces and pharynx, and extends by continuity from thence to the respiratory tubes; whilst the membrane lining the œsophagus may escape inflammatory action altogether, or become but partially implicated. These pathological relations, therefore, and the community of symptoms, will be kept in view, in examining into the nature of those lesions which affect these organs. The exact pathological conditions which exist in the throat and the air-passages in diseases of these parts have been until recently but imperfectly understood. Indeed, at the present day, several affections of the larynx and trachea are confounded by different writers, or their origin assigned to morbid conditions which do not exist; whilst, on the other hand, accumulated pathological facts show conclusively that there are other affections of the air-passages, whose characteristics and morbid relations are still involved in obscurity. To point out the seat and nature of one of these affections, to investigate its causes, and from a knowledge of its true pathology to establish correct principles of treatment, are among the objects of the present inquiry." (p. 24.)

Before, however, entering upon the special subject of his work, Dr. Green takes a brief survey of the principal pathological changes upon which the diseases known as chronic bronchitis, laryngitis, &c., are supposed to depend, with the object of affording his readers the means of appreciating his own views more fully.

The structural changes to which, according to Dr. Green's views, the mucous follicles of the throat are liable, are inflammation, ulceration, hypertrophy, induration, and tubercular degeneration, attended, in most of these conditions, by a greatly increased and vitiated mucous secretion. The disease of the mucous glandulæ may be *primary* and *uncomplicated*, and be limited entirely to the fauces and pharyngo-laryngeal membrane; or it may be *complicated* with *hypertrophy and induration of the tonsils*, and with *elongation* of the *uvula*. It may accompany, or be consecutive to other affections of the air-passages, and coexist with laryngitis, bronchitis, or pulmonary phthisis.

The literary history of the follicular disease of the pharyngo-laryngeal membrane is very barren. Dr. Badham is referred to by Dr. Green, as being the first who expressly called attention to it, under the designation of bronchitis. In the United States it first attracted attention as a sequel to an epidemic influenza.

"The first case of well-marked follicular disease, which came under my notice, was that of a clergyman, and occurred in the early part of 1832. During the preceding year, however, the attention of practitioners in different parts of New England had been called to the fact, that many clergymen in different sections of the country were seriously affected, and in some instances were wholly incapacitated for public speaking by 'a distemper of the throat,' which was characterized by symptoms of a peculiar nature and of unusual severity. By inquiries directed to this point, I have not been able to ascertain that any strongly marked cases of this form of disease had been observed prior to 1830. During this year it will be remembered that an epidemic influenza prevailed in this country, which not only extended over all the United States but spread throughout Europe, and so far as is known, over the whole civilized world. Whether this epidemic, or the causes upon which it depended, had any agency in increasing the frequency of the disease under consideration, or in changing its character into one of a more malignant nature, it is impossible now to determine. Certain it is, however, that while the influenza of this period was the precursor of epidemic cholera in some parts of the world, in many portions of the United States it was early followed by the above form of follicular disease. That this affection in its more aggravated form is of recent origin, or that it was formerly confounded with other diseases of the air-passages, we have the concurrent testimony of various writers who have alluded to the prevalence of the complaint. Professor C. A. Lee, of New York, who published in 1836, 'An Inquiry into some of the Causes of Disease among the Clergy,' remarked, under the head of 'Chronic laryngitis,' upon the alarming degree of prevalence which this disease had attained among that class of persons, and adds, 'It is but a few years since this disease was unknown almost by name, or if now and then a case did occur, it was generally of so mild a character, as to yield to very simple treatment.' For some time after the appearance of the disease in this aggravated form it seemed to be confined in its attacks to public speakers, and of these the clergy were the most frequent sufferers. Hence the affection was early called the 'Clergyman's Sore Throat.' It was soon found, however, that individuals of every profession, of all occupations, and of different ages and sexes, were liable to the disease. Of nearly four hundred cases that have fallen under my observation, only about seventy-eight, or one in five of this number, were in any way public speakers. When, however, the affection does occur in those persons who are in the habit of exercising the vocal organs by public speaking, singing, teaching, &c., it is always, for obvious reasons, attended with symptoms of a more aggravated nature, than when it appears under ordinary circumstances." (pp. 45-7.)

The first stage of follicular disease of the pharyngo-laryngeal membrane is characterized by subacute inflammation of the mucous follicles of the fauces and pharynx, and in its uncomplicated form, it is only by an extension of inflammatory action that those of the epiglottis, larynx, and trachea are affected. This is Dr. Green's opinion, but we think he will find it necessary to modify it, as it excludes the probability of the follicles of the epiglottis and larynx being affected primarily.

We subjoin Dr. Green's description of the progress of the disease:

"So insidious frequently is the onset of the disease, and so gradual its progress, that in some instances it will be found to have continued many months and to have made considerable advance before the presence of any prominent local symptom shall have called the attention of the individual to the existence of the affection. He then perhaps becomes aware of an uneasy sensation in the upper part of the throat, accompanied by a frequent inclination to swallow, as if some obstacle in the passage might be removed by the act of deglutition; or, more frequently, there is an attempt made, and often repeated, to clear the throat by a kind of screatus or hawking, and to relieve it of a sensation of 'something sticking at the top of the windpipe.' About the same time there is observed an alteration in the quality or *timbre* of the voice; there is experienced in the vocal organs a loss of power, and a hoarseness is present which at first is hardly perceived in the morning or after a full meal, but which is increased towards evening, and after speaking or reading longer or louder than usual. The mucous secretion, which, in a healthy condition of the glands is bland and transparent, becomes viscid, opaque, and adherent, and is increased in quantity. Frequently there is a slight soreness felt about the region of the larynx, but seldom is any cough present at this stage of the disease. In this condition the symptoms may remain for a long period, sometimes for years; nearly disappearing at times, and then again being greatly aggravated by vicissitudes of temperature, increased exercise of the vocal organs, and by various other morbific causes. If we inspect the throat and fauces during the progress of the above symptoms, we shall find the epithelium, which in the healthy state of the mucous tissue covers its surface, more or less destroyed; its absence being manifested by the slightly raw or granulated appearance which the membrane presents; the mucous follicles will be found hypertrophied, and will appear distinctly visible, especially those studding the upper and posterior part of the pharyngeal membrane. If the disease has been long-continued, a portion of the follicles may be found indurated, or, in some instances, filled with a yellowish substance, having a resemblance to and presenting the physical characters of tuberculous matter; whilst striæ of opaque, adhesive mucus, or of a muco-purulent secretion, may be seen hanging from the veil of the palate, or coating the posterior wall of the pharynx. As the disease advances, and the follicles, situated at the root of the epiglottis and in front of the arytenoid cartilage, and the still more numerous glandulæ of the laryngeal mucous membrane, become involved in the morbid action, all the above symptoms appear greatly aggravated; the hoarseness is much increased, and is constant; speaking or reading aloud is attended with great difficulty; and when continued for any period is followed by pain and increased soreness in the region of the larynx, and by a sensation of extreme languor, not only about the vocal organs, but throughout the whole system. In some cases, where the disease affects the glands situated in the ventricles of the larynx and near the vocal chords, the voice becomes completely extinguished; or, if by great effort, the patient essays to speak aloud, the vocal resonance is uneven, harsh, and discordant. In such cases, notwithstanding the situation and extent of the disease, there is seldom present any decided or troublesome cough; and, in this respect, follicular disease differs essentially from all other equally grave, laryngeal affections. Cases have fallen under my observation repeatedly, where the affection had advanced until the symptoms present indicated extensive disease of the follicles of the larynx and of the membrane covering the vocal ligaments; until the ulceration of these glands, situated at the root of the epiglottis, could be felt upon the laryngeal surface, and yet the patient would remain free or nearly free from a cough, notwithstanding an abundant acrid secretion, poured out by the diseased follicles, would occasion an incessant hawking to clear the upper part of the windpipe and the pharynx of this tenacious mucus." (pp. 50-3.)

It is of importance to remember, however, that the disease will commence and become inveterate without any symptom whatever being referred to the throat. The patient is teased by a constant irritating cough, his breathing is difficult, he snores loudly in consequence of the thickened membrane, and awakes suddenly from the short slumbers which constitute the sole repose he gets, and coughs up a viscid mucus; yet he will express himself as having never experienced any pain or uneasiness in the throat or larynx, and is surprised that such particular reference should be made to the condition of the throat, inasmuch as the seat of the irritation, as felt by himself, is at the root of the neck, about the point corresponding to the bifurcation of the bronchi. The symptoms vary in fact so much in different individuals, that it is evident a much more minute and accurate investigation is requisite, with a view to a proper classification of the different forms under which this follicular disease appears. The following case will serve to illustrate its origin and progress as the "clergyman's sore throat:"

"In June, 1841, Rev. Mr. S., of this city, aged 34 years, came under my care for an affection of the throat, which, for the four years preceding this period, had incapacitated him for public speaking. The disease came on much in the same way as its access is described to have been in the preceding cases. Whilst engaged in his official public duties, he had observed for some time an increasing irritation and soreness about the fauces and throat, accompanied by a hoarseness and a frequent inclination to clear the voice when speaking. These symptoms, which ordinarily passed off after a few days of rest, were disregarded until his voice failed, and he suddenly broke down under his pastoral labours. Finding himself entirely unable to discharge his professional duties, although still possessing a good degree of general health, and after resorting to various measures to restore to a sound condition his vocal organs, all of which proved ineffectual, he resigned his ministerial charge, and sailed for Europe; cherishing the hope—in which his numerous friends ardently participated—that the sanative influence of a sea-voyage and a foreign tour would prove efficient in removing his most troublesome and perplexing malady. After an absence of many months he returned to his home, invigorated by this long relaxation from his duties, and although there still remained some sensibility of the larynx, and a slight huskiness of the voice, yet he felt that with due caution he might with safety gradually resume his public duties. A single attempt, however, to speak in public soon after his return, entirely dissipated this hope. His voice again gave way; hoarseness, with every other morbid symptom which previously attended his disease, returned in a still more aggravated form than at first. In this condition his case remained—marked by an occasional remission of the symptoms, but attended by no permanent improvement—until the period above mentioned, when, as I have stated, he came under my care. His condition at this time was as follows: His general health, although impaired by the long-continued local disease, was still very good; yet his countenance exhibited a sallow hue, and was marked by that care-worn and anxious expression which I have often observed in those cases of tuberculous sore throat which have been protracted through a long period of time. Constant hoarseness was present, and his voice when uttered aloud was rough and hollow. Speaking was accomplished with difficulty, and if continued, as in ordinary conversation, for a short time only, was followed by soreness and increased hoarseness, and, sometimes, for a short period, by complete aphonia. His throat, on being inspected, presented an enlarged and cavernous appearance; as if the pillars of the fauces and the pharyngeal muscles had become atrophied, or had been wasted away by disease in a manner greatly to enlarge the posterior fauces. The mucous membrane lining these parts was covered by diseased follicles, some of them greatly enlarged and indurated, others slightly hypertrophied, and filled with a semi-fluid substance resembling tuberculous matter. On pressing down the tongue, the epiglottis could be seen standing above its base, erect and œdematous; its edges red and slightly ulcerated; whilst a vitiated mucous secretion was being constantly poured out from the diseased glands, occasioning an incessant hawking to relieve the throat of this cause of irritation. Pressure upon the thyroid cartilage increased the pain and soreness, which were constantly felt in the larynx. This last symptom, together with the permanent hoarseness and the partial extinction of voice, was plainly indicative of the mucous follicles of the ventricles of the larynx and of those around the chordæ vocales being involved in the disease. Yet there was no cough present, nor could a rigid examination of the chest detect any morbid alterations in the pulmonary organs. All mental excitement affected him injuriously; it had a tendency invariably to aggravate the local difficulty, although no effort whatever might be made to exercise the vocal organs. The tongue was coated, his pulse seventy-six in the minute, and quite feeble." (pp. 58-61.)

The subjoined illustrates the progress of the affection in a barrister:

"K. H. E., Esq., a lawyer of eminence in this city, aged 38 years, suffered from an attack of acute bronchitis, in April, 1840. Under the most active treatment he recovered from the disease, and resumed his professional duties. In

1841-2 he was a member of the Common Council, and, in addition to the duties of a full practice which necessarily involved much public speaking, he was frequently engaged in the exciting debates of the honorable body of which he was a member. Early in 1842 he began to be sensible of a slight huskiness of the voice, and of an uneasy sensation in the throat after public speaking. These symptoms would all subside after a little rest, but only to be renewed at each subsequent public exercise of the vocal organs. It was observed that this hoarseness gradually increased, and that the irritation about the throat impelled the individual to make frequently repeated efforts at hawking, as if to remove some obstruction from the larynx. Being in attendance upon his family during the progress of these symptoms, I had frequent opportunities to inspect his throat, and I observed that the follicular glands of the isthmus of the fauces and of the superior portion of the pharyngeal membrane were slightly hypertrophied, and were pouring out an altered and increased secretion. Believing on his part that these morbid symptoms would pass away, no special attention to his case was required or given until the latter part of July, 1842. At this time a permanent hoarseness was present; the voice was rough and uneven, with a constant irritation and a sensation of soreness in the laryngeal cavity; symptoms that were all greatly increased by every effort made and continued to speak or read aloud. The diseased follicles now presented a very different appearance from that which they had exhibited a few weeks before. The posterior fauces and pharyngeal membrane were studded with elevated tubercles, with inflamed bases, or granulations of different sizes, like pustular inflammation, bearing a marked resemblance to the papulæ of varioloid. The most pendant portion of the uvula, which was greatly elongated, was now covered by similar diseased follicles." (pp. 64-5.)

In some examples, the disease goes on until the symptoms attain so great a degree of intensity as to simulate tubercular disease of the lungs. Of this by no means uncommon circumstance, the subjoined case is an instructive example; it is that of a merchant, aged 37:

"Coming on in the insidious manner that has been described, the disease had made considerable progress before his case was deemed of sufficient importance to require medical treatment. At length he placed himself in the hands of some homœopathic physicians of this city, under whose treatment he remained for two or three years. In the meantime the disease, of course, continued to advance, until extensive ulceration of the pharyngo-laryngeal follicles had taken place. Still placing his confidence in this 'fabric of a vision'—Homœopathy, he visited Paris, and put himself under the care of the celebrated Hahnemann, who treated his case for three or four months; but with as little success as had attended the prescriptions of his satellites in New York. Discouraged at last, or losing confidence in the plan of treatment proposed by Hahnemann, he came back to New York, and soon after his return placed himself under my care. His general health at this time had become much impaired, doubtless through the long-continued influence of the local disease. The whole throat, which presented an atrophied and cavernous appearance, was studded with diseased follicles, some of which were greatly enlarged and vascular, or were filled with tuberculous matter; whilst others were broken down and destroyed by ulcerations. The uvula was elongated, and the epiglottis, which could be seen above the back of the tongue, was erect and œdematous, and its circumference was serrated with ulcerations. On examining the epiglottis with the finger, an extensive and deep ulcer could be felt at its base, on the laryngeal surface of this cartilage; whilst the pain in the larynx, the soreness experienced on pressure over the thyroid cartilage, together with the constant hoarseness and irritation in the laryngeal cavity, showed conclusively that the ulcerations had extended to the vocal ligaments. The patient complained of a dull pain, and a sensation of great weakness under the sternum; there were present also, a cough, emaciation, erratic pains in the chest, and other constitutional symptoms that indicated the presence of tubercles in the lungs; but, notwithstanding many of the rational symptoms of phthisis were present, a careful examination of the chest was made, without detecting any structural lesions in the pulmonary organs. Bronchial irritation, however, existed to a considerable extent." (pp. 69-71.)

We observe here that the plan of treatment adopted by Dr. Green was so eminently successful in this case, that his patient was ultimately restored to robust health. We could add also, if our space would permit, another case presenting all the rational signs of ulceration of the larynx, with the voice reduced to a rough whisper, in which the repeated application of a strong solution of crystallized nitrate of silver (forty grains to an ounce of distilled water) to the cavity of the larynx, combined with other treatment, was perfectly successful in restoring the patient to health and the full performance of his duties as a clergyman. Another example of a merchant is given, in whom the voice had been no louder than a whisper for fourteen months, and who was treated by the nitrate with the same success: but we are anticipating our subject.

In the examples of follicular disease above detailed, no difficulty of deglutition was experienced; in some, however, this is a prominent and urgent symptom. A married lady, aged 32 years, exhibited some of the earlier signs of phthisis, to which she had a hereditary predisposition, but from which, however, she recovered. Some few months afterwards—

"She began to experience a slight uneasiness in her throat, accompanied by soreness, and a constant desire to clear the passage; symptoms which were soon followed by hoarseness, a slight cough, increased tenderness in the laryngeal region, and a difficulty in swallowing. So urgent, indeed, had this latter symptom become, that for several weeks preceding her visit to New York, she had found it very difficult to swallow her food, unless taken in a liquid form, or made soft by careful mastication. I saw her on the 3d of September, and found her pale, feeble, and emaciated; pulse languid, voice hoarse and raucous, and at times reduced to a whisper; cough frequent, and accompanied by a slight expectoration of viscid mucus. She complained of a difficulty of deglutition; a sense of smarting in the gullet, and soreness whenever pressure was made over the thyroid cartilage. On inspecting the throat, the enlarged cavity of the posterior fauces was found covered with diseased follicular glands; some of them in a state of ulceration, and others were filled with a puriform matter. About one third of the epiglottis was in sight; its circumference and lingual face were extensively ulcerated, which accounted for the pain and difficulty which the patient experienced in deglutition; the uvula was considerably elongated, and the curtain of the palate was pale, relaxed, and œdematous." (pp. 87-8.)

*Follicular disease of the tonsils.* In some patients the tonsils are the principal seat of the disease. In the following example of a merchant, aged 25, a prominent homœopathic physician of New York had treated the patient for three or four months, and had assured him three days only before he consulted Dr. Green that every vestige of disease was removed from his throat!

"The whole outward or rational symptoms manifested by this patient are those which indicate an advanced stage of pulmonary phthisis. He is pale and greatly emaciated; countenance presents an anxious and haggard expression; he has copious night-sweats, dyspnœa, and a constant harassing cough, which is attended by a free expectoration of muco-purulent matter; the voice is thick and hoarse, and deglutition is performed with much difficulty. The dyspnœa is greatly increased whenever the patient lies down, and for many weeks no other individual has been able to sleep in the same room with him, on account of the laboured and stertorous breathing which is constantly present during sleep. The throat, on inspection, is found to be apparently entirely filled up with two enormously large and ulcerated tonsils (see Plate V), between which the uvula, which is also hypertrophied, appears wedged in, like the keystone of an arch. No portion of the pharyngeal membrane can be seen, as the morbid mass completely blocks up the view. How respiration can be carried on, or deglutition performed, with the throat in this state, it is difficult to imagine; and still more difficult is it to comprehend how a learned physician could have pronounced such a throat to be in a healthy condition! On exploring the chest no morbid signs are detected, except such as indicate the presence of some degree of bronchial irritation. But there is soreness in the region of the larynx, and pain, which is increased by pressure over the thyroid cartilage." (pp. 92-4.)

In this case the tonsils were of course removed, and the operation laid the pharyngeal follicles open to view in a hypertrophied and ulcerated condition, to which the solution of nitrate of silver was applied. Ultimately the patient was restored to perfect health. Dr. Green remarks:

"In the preceding case, the pharyngo-laryngeal affection may, in some respects, be considered as having been complicated with bronchial disease; but the inflammation of the bronchial vessels, which undoubtedly had existed to a considerable extent in the earlier stages of the disease, was not present when the patient came under my care. The effect, however, of the inflammation was still remaining, and this consisted in a dilatation of some of the bronchial tubes. This alteration of structure has led to a great error in diagnosis; for, misled by the external or rational signs—the emaciation and dyspnœa, the night-sweats, and the free muco-purulent expectoration of the patient—several physicians, both in this city and Boston, who had examined the case, had mistaken the bronchial dilatations for tubercular cavities, and had pronounced it to be one of confirmed phthisis. That it was not tuberculous disease, time has proved, by establishing the permanency of the cure. It is now over two years since the patient was under treatment, during this period he has passed through two severe winters, in which he was constantly engaged in business; and yet he has been, and still remains in excellent health." (pp. 95-6.)

Disease of the *follicles of the uvula,* together with hypertrophy and other morbid changes, is noted by Dr. Green as being seldom wanting in a large majority of cases like those detailed. The influence of elongation of the uvula on the pulmonary mucous membrane has been long known to the discriminating practitioner. The irritation it excites induces, after a period more or less prolonged, many of the rational signs of phthisis. The following case illustrates the morbid effects that may be produced by it in the apparent absence of all other causes:

"In 1841, E. B., aged 26, a merchant of this city, suffered severely from follicular laryngitis. During this year he left the north, and spent a part of the inclement season in one of the southern States. But his disease continuing to increase, he came back to New York, and after his return, placed himself under my care. At this time there was debility, loss of flesh, with cough and hoarseness, and a constant irritation about the throat: an examination of which revealed an extensively diseased condition of the follicles of the fauces and of those of the pharyngeal membrane. His uvula was moderately elongated, but not to that extent that seemed to require excision. Under the use of those topical and general remedies which are comprised in the plan of treatment that was employed in many of the preceding cases, this patient was restored to a good degree of health; the hoarseness and soreness of his throat disappeared, the mucous membrane assumed a healthy appearance, and he regained strength and flesh, so that he was enabled again to attend to his business. His cough, however, never left him, but, on the contrary, notwithstanding the employment of many of the ordinary remedies for its removal, continued to increase, and was attended, after a while, with a free expectoration of muco-purulent matter. The occurrence, at length, of erratic pains about the chest, with debility and other unfavorable symptoms, led the patient and his friends to apprehend the presence of confirmed pulmonic disease. At this crisis, after having resorted to various remedial measures without benefit, I proposed the excision of the patient's uvula, which, although but moderately elongated, was evidently a source of irritation. To this the patient acceded; the uvula was removed, and from that hour the cough ceased, the pain in the chest, and every other indication of thoracic disease which had been present, soon passed away; and from that hour to the present the patient has been free from all pulmonic disease." (pp. 103-4.)

The complication of follicular disease of the pharyngo-laryngeal membrane with laryngitis and bronchitis is frequent, as might *a priori* be expected. There is at first an expectoration of transparent, adhesive mucus. As the disease advances this discharge increases in quantity, and is characterized by the presence of opaque or albuminous matter, commingled with the more transparent liquid mucus. Emaciation, debility, night-sweats, and even slight hemoptysis may also ultimately supervene. In such cases, Dr. Green has succeeded in restoring his patient to a fair degree of health and strength.

The complication of follicular disease with phthisis pulmonalis is, according to Dr. Green, of no unfrequent occurrence, and renders all the symptoms more harassing and distressing; its cure ought therefore to be attempted:

"When coexisting with tubercular phthisis, the cough, the dyspnœa, and all the characteristic symptoms of the latter disease are greatly aggravated, and the affection passes through its stages and reaches its fatal termination much sooner ordinarily than when its progress is unattended with follicular disease of the pharyngo-laryngeal membrane. Although but little can be expected from any attempt made to prevent the final *dénouement* in these cases where disorganization of the lungs has occurred, yet, in many instances, the harassing cough, the difficulty of deglutition, and the dyspnœa which are often present in this combination of disease, are signally relieved, and the sufferings of the patient greatly mitigated, by the employment of topical medication upon the diseased laryngeal surface. So marked, indeed, has this relief been in some cases which have fallen under my observation, even when tubercular cavities have been present, as to awaken in the minds of the patients and their friends strong hopes of their final recovery. In other instances, and these have been not a few, where follicular laryngitis has preceded thoracic disease, I have seen the symptoms of incipient pulmonary affection rapidly disappear after the removal by topical medication of the primary follicular disease." (pp. 119-20.)

Dr. Green details an interesting case of phthisis, in which the treatment was singularly successful in mitigating the disease. Cauterization of the fauces, glottis, and cavity of the larynx was continued every day, on each alternate day, for several weeks; counter-irritation over the superior portion of the spinal column was kept up, and along with the alterative remedies prescribed at the commencement of the treatment, the patient was put upon the use of general tonics. Under this plan of treatment the improvement of his health was marked and rapid. He was able to swallow solid food without pain or inconvenience, his strength increased, his voice was improved, he had regained several pounds of flesh, whilst every evidence of ulceration of the throat had disappeared; and all this mitigation of the disease took place notwithstanding the symptoms which have been enumerated,—in connexion with the morbid physical signs which auscultation revealed—indicated the presence of extensive tuberculous disease of the lungs. These latter indications increasing, he died of pulmonary phthisis.

Malignant follicular disease of the œsophagus is noticed by Dr. Green, and two cases related, but they present nothing of special interest.

*The pathological anatomy of follicular disease* is next discussed by Dr. Green; we do not find much addition to our existing information. Hypertrophy may occur, with or without induration of the follicles. When the mass of glands which are aggregated in the tonsils suffer from long-continued inflammation they become indurated. The induration is not generally of a malignant character, but depends, Dr. Green observes, upon the presence of a deposition of fibrin, which during the process of inflammation has been lodged in the cavity of the follicle or in the interstitial substance with which it is surrounded. The matter thus deposited very readily becomes vascular, is supplied with blood-vessels, and at length becomes organized; but this process takes place to a limited extent, for when excised the enlarged tonsil seems to possess but little sensibility.

Calcareous deposit sometimes occurs, and we believe it is much less rare than many suppose. Patients will cough or exscreate little calculi, which have evidently come from the tonsils.

The secretion of these muciparous glands will become morbid, and, like that in other mucous membranes in a state of inflammation, will assume an acrid and irritating quality, and thus the investing membrane is not only stimulated, but the disease is extended to adjoining follicles. Dr. Green remarks also, that hemorrhages will take place from them, and in illustration, mentions the case of a young gentleman, who for a long period has suffered under follicular disease of the throat, and who, without manifesting any symptoms of phthisis, has had repeated hemorrhages from the pharyngeal mucous membrane. The spot indeed can be distinctly seen from whence the blood exudes, and yet no abrasion of the surface, nor the rupture of any vessel can be detected. One or two similar examples have come under our own notice in the persons of inveterate smokers.

Dr. Green states that the secretion of a fluid, possessing all the sensible and chemical properties of pus, is the frequent result of disease of the pharyngo-tracheal follicles. When the disease in these glands has passed on to the stage of ulceration, the purulent secretion is marked and abundant, and its source is apparent; but it sometimes occurs where no structural lesion, either of the follicles or of the lining membrane, can be detected. Generally, however, when purulent secretion takes place in follicular inflammation, the glands are found to be more or less in an ulcerated condition; in many instances they may be seen in the posterior fauces and on the pharyngeal membrane, some of them ulcerated, others distended with purulent matter. The fluid, likewise, which is poured out from the follicles of the tonsils, when these glands are hypertrophied and inflamed, is not unfrequently of a purulent nature.

The deposit of tubercular matter in the substance or on the surface of the larynx, epiglottis, or trachea is denied by M. Louis. Dr. Green combats this doctrine, and quotes Andral, Carswell, Williams, and Hope, in support of his own observation, that "in enlarged mucous follicles of the upper air-passages, and imbedded in the tissues of these parts, were morbid deposits, presenting all the physical characters of true tuberculous matter." He thinks that Dr. Carswell's supposition that tuberculous matter may often be secreted upon the free surface of the membranes of these parts, but that not being entangled or confined in any mucous crypt, it is removed as soon as it is formed, reconciles the conflicting statements on the subject.

Ulceration of the follicular glands is a frequent termination of long-continued irritation. After the irritation has persisted for some time, the engorged follicle presents a small ash-coloured point, which is surrounded by an inflamed base, and has red and slightly-elevated edges. In follicular disease, these ulcers, which ordinarily spread slowly, are generally first observed about the arches of the palate and on the back of the pharynx; they next attack the laryngeal face of the epiglottis, and the epiglottic glands situated at the base of this cartilage, and spreading by continuity, they in some instances invade the mucous follicles in the ventricles, and around the chordæ vocales. Indeed there is no part of the larynx and trachea that may not be the seat of ulceration. In their early stages, ulcerations of the mucous glandulæ are small and superficial; continuing for a long time, not only are the glands destroyed, but the mucous, the sub-cellular tissues, and even the cartilages themselves may become involved in the ulcerative process.

Thickening of the mucous membrane of the pharynx, &c., is an early change in the progress of follicular disease, but eventually an opposite state of things takes place, for not only are the surrounding engorged membranes unloaded, and their increased thickness removed, but the sub-cellular tissues and the pharyngeal muscles become atrophied; in part, probably, from the increased absorption which has been set up; and we then have, on inspection, those enlarged or cavernous throats, so frequently observable in long-continued follicular disease, and to which allusion has more than once been made.

*Causes of follicular disease.* These are either predisposing or exciting. Dr. Green notices first as a predisposing cause, a hereditary tendency to the disease. That this exists in families is, he observes, a well-established fact.

"At the present time, I have under treatment three brothers, clergymen, who have been compelled to relinquish their official public duties on account of follicular disease of the throat; and whose mother, now over eighty years of age is labouring under the same affection. In another instance, coming also under my observation, four members of the same family, with one of the parents, were the subjects of follicular disease; and among my notes a large number of cases are recorded, where two and three members of the same families have been treated for this affection." (pp. 158-9.)

A strumous diathesis, and all those influences which induce a cachectic state of the system, predispose to the affection, and among these influences no one is more prominent than impure air, such an atmosphere, for example, as clergymen, teachers, lecturers, and other public speakers, are frequently compelled to breathe in crowded and ill-ventilated churches, lecture-rooms, &c. Severe and protracted labour, conjoined with anxiety about temporalities, is a very frequent cause of the disease amongst clergymen, especially "that numerous class who, settled in the towns and villages of the country, are compelled to sustain themselves and their families upon salaries which, with the practising of a most rigid economy, are barely adequate to supply them with the necessaries of life."

The male sex is more predisposed to follicular disease than the female. Louis has already established numerically, that a much greater proportion of males suffer from ulceration of the trachea, &c., in phthisis, than females. Of one hundred and ninety subjects carefully examined by him, one half of the males, but only one fourth of the females, presented ulcerations of the trachea; a similar but rather less proportionate difference was observed with reference to ulcerations of the larynx and epiglottis. Louis found that there was a greater difference in the frequency of ulcerations of the bronchi in the two sexes. Forty-seven phthisical subjects were examined by him; nineteen were women, of whom five had bronchial ulcers, or a proportion more than one fourth; whereas, seventeen of thirty men were thus affected, or more than one half, so that Louis's data do not bear out Dr. Green's observations, that the upper portion specially of the respiratory tube is more affected in men than women; on the contrary, we find the proportionate preponderance of males affected by ulcerations of the respiratory mucous membrane diminishes from the bronchi upwards, it being least with regard to the larynx and epiglottis. There are various reasons which might be assigned for this preponderance. The respiratory system is more highly developed in males; is more exposed to the ordinary causes of inflammation; tobacco-smoking in particular, a habit almost exclusively masculine, is more likely to induce in males pulmonary disease of the kind under consideration, by the irritation excited in the pharyngo-laryngeal tissues. The vocal organs are also called into unequal or prolonged exercise by the avocations of life more generally in the male sex than in the female.

The age of the largest number of patients treated for follicular laryngitis by Dr. Green was from 25 to 35; in very few was the disease manifested at an earlier period.

Among the exciting causes, Dr. Green enumerates an attack of influenza or of eruptive fever, but particularly the habitual use of tobacco:

"As an exciting cause, the use of tobacco, in my experience, has proved a powerful agent in the production of follicular disease of the throat. Acting as a stimulant, directly and constantly, upon the mucous follicles of the fauces and throat, and greatly increasing, as it does, the secretion of these glands, its employment, as we should conclude, *a priori,* must have a direct tendency to develop the disease, especially if a predisposition to the affection exists; hence it has occurred to me to notice, that of a great number of cases of throat-ail, which during the last year or two have come under my observation, a large proportion of them have taken place in individuals who had been, or were at the time, in the habitual use of tobacco. My attention has been called more particularly to this subject, from having noticed, several years ago, some observations on the use of tobacco in laryngeal and bronchial affections, by an eminent surgeon of this city. After having alluded to the almost universal use of tobacco in the countries of Northern Europe, he observes—'In one very fatal and distressing form of disease, to wit, laryngeal phthisis and bronchitis among public speakers, the fact is very clearly established, that the moderate habit of smoking, by the drain it accomplishes, and its anodyne qualities, has been eminently useful, at least as a preventive, of that peculiar malady so frequent in the United States, especially among the clergy.'* From this opinion of my distinguished countryman and friend I am compelled to differ entirely by the statistical facts which I have obtained on this subject. Not only has the use of tobacco, in any and all its forms, proved in my experience an exciting cause of laryngeal disease, but where its employment has been persisted in during the treatment of any case, I have found it impossible to restore such to perfect health." (pp. 175-7.)

Dyspepsia has been mentioned as a cause of the disease, but Dr. Green is disposed to consider it in many cases rather as an effect; and that it is induced by the vitiated secretion from the follicles carried into the stomach with the food and drinks of the patient.

The regular use of the voice is not to be considered as an exciting cause, as Dr. Green found few barristers or auctioneers to be affected, but he thinks that an irregular or sudden and violent use of the vocal organs will bring it on, especially when a predisposition exists. Thus clergymen suffer rather than barristers, because their duties lead them to prolonged public speaking on one day of the week only, while during the remaining six the vocal organs are nearly quiescent.

*Symptoms of follicular disease.* These our readers will have already become acquainted with from the details of the cases quoted above. Perhaps, however, it will be useful to recapitulate them.

In some cases the disease will have made considerable progress before the attention of the sufferer is directed to the throat, but—

"Ordinarily, however, soon after the mucous glandulæ have taken on a morbid action, there is perceived in the region of the fauces an increased mucous secretion, and an uneasy sensation in the gullet or upper part of the throat is observed, attended by a frequent desire to swallow, as if some object sticking in the passage might be removed by the act of deglutition; or, more generally, repeated attempts are made by hawking to clear the throat, and allay the irritation; all which difficulties are considerably augmented by every continued effort made to read aloud, to sing, or to speak as in ordinary conversation. If the secretion from the mucous follicles of the throat be examined at this period, it will be found to be altered in its character, being adhesive, and, in some instances, of an alkaline quality, and proving to be, by its effect on the mucous membrane, of an irritating nature." (pp. 178-9.)

Alterations in the tone of the voice now become perceptible, particularly if the individual be accustomed to sing or speak in public, and is more perceptible in the evening than in the morning.

"On inspecting the throat, the fauces and the posterior wall of the pharynx will appear redder than natural, and the mucous membrane covering these parts will be deprived of its epithelium, injected, and studded over with enlarged mucous follicles. Sometimes, if the disease is recent, these glands will appear quite minute, and will be distinctly apparent only when the pharyngeal cavity is exposed to a full light. In other instances they will have attained a size sufficient to give a rough or granular appearance to the whole surface of the fauces, while the viscid tenacious mucus which is poured out by these follicles in their morbid state may be seen coating the membrane, or appearing in patches, or marking its surface with white or yellowish-white striæ. In some cases several of the enlarged and morbid cryptæ will become confluent, and, uniting, form angry-looking tubercles, of the size of a split pea, which may be seen on the posterior wall of the pharynx. In others, again, a deposition of textural matter takes place, and the follicle becomes indurated and permanently enlarged; or it may be distended with pus, or with a morbid secretion, which

* Travels in Europe and the East. By Valentine Mott, M.D. pp. 83-4.

will exhibit all the physical properties of tuberculous matter. If the affection has continued for some time, we shall frequently find some of the diseased follicles in an ulcerated state; these are generally first observed about the palatine arch, the posterior wall of the pharynx, and along the border and on the laryngeal face of the epiglottis. In the first stage these ulcers are small and superficial, appearing in the form of ash-coloured patches, surrounded by an inflamed and slightly elevated base. Continuing, they at length destroy the mucous follicles, and sometimes involve not only the mucous, but the sub-cellular, tissues in their progress. Accompanying the above symptoms, there is often found œdema and elongation of the uvula, and in many instances hypertrophy of the tonsils." (pp. 179-81.)

In the incipient stage of follicular laryngitis, of the uncomplicated form, there is seldom much cough present. The irritation that is felt in the larynx, and which is caused by the increased and vitiated secretion from the diseased follicles, is generally relieved for the moment by hawking in this stage of the affection. As the disease advances, however, and the glandulæ of the larynx and trachea become involved in the morbid action, a cough will steal on, which, from being slight at first, is at length severe, and in most cases is attended by a free tenacious expectoration. In two examples under our care, and in which we had no reason to suspect laryngeal ulceration, the cough was incessant, harassing the patient both night and day, and was by far the most prominent symptom. Nor was the expectoration in one of these cases "free:" indeed the nature and amount will depend entirely upon the extent of the bronchial irritation excited; so that the diagnosis between the earlier stages of tuberculous disease and a follicular affection, founded by Dr. Green upon the circumstance that, in the latter, there is a free tenacious expectoration, while, in the former, it is only a trifling amount of transparent frothy fluid, is somewhat apocryphal. We are inclined to think that those gray firm pellets of consolidated mucus, occasionally expectorated by individuals otherwise healthy, indicate the first stage of follicular disease. We fully coincide, however, in the subjoined opinion:

"In another respect, these two diseases are essentially different. That peculiar mental condition incident to pulmonary disease,—by which the spirits of the patient are buoyed up, and hope often continues bright to the last,—is well known. The reverse of this obtains in follicular laryngeal disease. In this latter affection *mental depression* is, to some extent, so universally present, particularly where the affection has been protracted, that I have been led almost to consider it a characteristic of the disease." (pp. 183-4.)

After the disease has extended downwards, and ulceration within the larynx is established, the cough becomes much more severe, and the voice more altered. The tone of the voice is in some degree a guide to the seat of the irritation: if the ulcerations are confined to the follicles about the tonsils, the veil of the palate, and the pharyngeal membrane, the timbre of the voice is not ordinarily much changed; incomplete disphony sometimes exists, or, in other words, the sounds are merely obscured, or imperfectly articulated. But let the ulcerations extend below the epiglottis, and the hoarseness is greatly increased, the voice loses its power, and should the mucous glands within the ventricles and around the vocal chords become involved in the morbid alteration, it is reduced to a state of complete aphonia, and a harsh whisper, which is merely an articulation of the ordinary respiration, alone remains.

There is one symptom, which we think Dr. Green does not notice, but which the practitioner will find a frequent accompaniment of this follicular disease, and that is, a loud snoring during sleep, far louder than the ordinary snore of the comfortable sleeper. This will be more particularly heard in hypertrophy of the tonsils or uvula, and in thickening of the pharyngo-laryngeal membrane.

When the epiglottis, and particularly its inferior border, is extensively ulcerated, great difficulty of deglutition, with pain, and sometimes dyspnœa, is experienced. There is an expectoration of an opaque secretion, which seems to come from the very top of the throat, and this frequently increased after eating, and is sometimes tinged with blood; or small masses of dark, almost coagulated blood, will be mingled with the sputum. These ulcers often escape observation, and keep up irritation after the other portions of the fauces are restored to their normal condition. To detect them, it is necessary to draw the whole mass of the tongue forward with a broad curved spatula, which we shall more particularly describe under the head of treatment. Dr. Green attaches considerable importance to the characters and situation of these ulcers as aiding the diagnosis, with reference to the condition of the inner surface of the larynx, although no very specific information is given to his readers on this important point.

"If the follicles, situated along the border and on the laryngeal face of the epiglottis, become ulcerated, I have observed that this organ, which in its normal state is slightly crescentic, loses this form, and appears flattened like the tongue; it is, moreover, enlarged and thickened, and its border may be seen frequently serrated by the erosions. When that cluster of follicles, which constitutes the epiglottic gland, becomes the seat of ulceration, the epiglottis will assume nearly an erect form, and be found incurvated, or its crescentic shape considerably increased; and when this lesion has extended to the numerous glandulæ of the ventricles, and to those around the chordæ vocales, the above alteration of the form of the epiglottis will be still greater; its lateral edges will then be found rolled in towards each other, so that the organ will present nearly a tubular form, with its convexity towards the dorsum of the tongue.........Another aspect, indicative of organic changes, which the epiglottis presents, is that lesion which Dr. Stokes calls the leaf-like expansion of the epiglottis. In one instance observed by this writer, the epiglottis was thinned and singularly elongated, and its form so altered as to present the shape of a battledore, the narrow extremity being next the glottis. In this instance, the lesions of the epiglottis coincided with double perforating ulcers of the ventricles." (pp. 188-90.)

*Treatment of follicular disease.* This is divided into two parts, topical and general.

With regard to the topical treatment of pulmonary, pharyngeal, or tonsillar disease little need be said; inhalation, insufflation, lotions, and the application of the solid nitrate of silver to the tonsils, uvula, and pharynx, are the well-known means adopted for this end. The application of nitrate of silver to the cavity of the larynx is not, however, to be classed amongst these ordinary methods; and the practice of it by Dr. Green seems to have been received with such incredulity in the United States, that Dr. Green has thought it necessary to multiply evidence as to the fact that he has introduced a strong solution of nitrate of silver within that cavity. Trousseau and Belloc are supposed, by Dr. Green, to have been the first to prescribe and employ topical medication in chronic laryngeal disease. They found a solution of the nitrate of silver, in the proportion of two drachms, or sometimes, four drachms, to an ounce of distilled water, to be the most efficacious and harmless application. Two methods were adopted by them: the one was to saturate a small sponge attached to a bent rod of whalebone, and to manipulate so that the solution be expressed into the larynx; the other was to use a small silver syringe, with a tube suitably adapted for effecting the same object. Dr. Green, however, several years before the appearance of Messrs. Trousseau and Belloc's book, had instituted experiments, and came to a similar conclusion. Without wishing to disparage in the slightest degree the labours of our Gallic or American brethren, we subjoin a case which shows that above thirty years ago, our own countryman, Sir Charles Bell, successfully adopted the method of treatment so fully illustrated (and we think established too) by Dr. Green:—the italics are our own.

"Third case of disease of the larynx (from 'Surgical Observations, being a Quarterly Report of cases in Surgery;' by Charles Bell. London, 1816, p. 34). *Fanny Murray*, æt. 25, Regent's Ward. A few days ago Dr. Southey sent this girl, then an out-patient, to be examined, and as a case deserving to be watched. I immediately recognised the symptoms in the case of Mellon. She spoke in a whisper, scarcely audible, and with great effort; when desiring to be heard, she made an effort like a cough; *I put my finger into the glottis, and felt it rough.*

"*April* 18*th*. I was requested by Dr. Southey to visit his patient. She had been brought into the hospital very unwillingly, and is in imminent danger of suffocation. Her sense of danger has led her to the hospital. Since Christmas she has been ill; she was at that time attacked with cold and sore-throat, and from the beginning she could speak only in a whisper. Her voice has never returned, and at present her whispers are scarcely audible. She coughs in a very singular manner; she says it is an inward cough; it is the exertion of coughing without the sound. This I imagine to be produced by the want of action in the slit of the glottis, by which the air should be somewhat impeded, and thereby more forcibly sent through the glottis. She has pain at the pit of her stomach and in the loins, which I attribute to the exertion and fatigue of coughing. For these three nights she has not been able to lie down in bed. She expectorates a good deal: it is mucus and pus. Pulse 63, br. 42 in the minute. Her breathing has a harsh, sawing sound.

"*Evening of the* 18*th*. The hospital attendants becoming alarmed at the condition of this woman, I was sent for at eleven o'clock. She was sitting up in bed, breathing with much difficulty; but her countenance was of a red colour, the violence of the fit had subsided, and the blueness had been succeeded by redness and fulness. Dr. Southey came in. We wished her to swallow: she tried a little broth; much of it went into the windpipe, and she had a great struggle in recovering. We concluded that the epiglottis was eaten away by ulceration. I proposed mercurial fumigation, but my colleague did not expect benefit from it, as the patient had been three weeks under salivation without the slightest advantage. Having ascertained, by *putting my finger over the root of the tongue into the glottis, that it was rough and irregular with ulceration, I proposed to touch the surface with the argentum nitratum.* It was considered hazardous, but something was necessary, and I was confident that the application would allay irritation. I made a small pad of lint, and attached it to the ring of a catheter wire, and bent the wire so as to pass over the root of the tongue and epiglottis; *I dipped the lint in a solution of twenty grains of the caustic to half an ounce of water, and touched the glottis with it in this manner.* With the fingers of my left hand I pressed down the tongue, and stretched the forefinger over the epiglottis; then, directing the wire along my finger, *I removed the point of the finger from the glottis and introduced the pad of lint into the opening, and pressed it with my finger.* On withdrawing the lint, instead of coughing, she began to speak more audibly than usual, and had neither cough nor spasm from this rough operation. I repeated the application four times, and her breathing was sensibly better when I left her.

" 19th. This morning, at the visit, I found her considerably better. I touched the chink of the glottis again with the some solutions. I recommended issues to be made on the sides of the throat.

"20th. The issues not made. Last night it was reported to me that she was not so well, but this morning I find her remarkably better. I again touched the glottis with the solution of caustic, notwithstanding she complains of soreness of mouth from the former applications; it had the same good effect, improving her breathing as formerly. A pill of belladonna is ordered, and she is to smoke the stramonium: she is to put her feet and legs in the nitro-muriatic bath twice a day.

"22d. She has had the acid bath to her feet several times, and has been twice in the slipper-bath, with the acid more diluted, and the fluid reaching her loins. She speaks with more force, coughs less. Still she does not swallow better.

"26th. At Dr. Southey's request I again touched the glottis with the caustic solution. *I applied it as strong as it could be made.* The girl is frightened, but better.

"29th. *The girl is remarkably better;* she has taken the bath twice a day; her gums are sore, swollen, and of a dark red colour. Her countenance is much amended; she breathes without noise, and can swallow better than she did.

" *May* 4th. She can swallow better; she breathes easily, but she is full of complaints and uneasiness. Her mouth and throat are much inflamed, the gums dark coloured and spongy. She is to take the nitro-muriatic bath every second night.

" 5th. The bath to be altogether omitted. Dr. Scott visited this patient; he never saw the mouth so sore from the operation of this remedy.

"30th. I examined this girl yesterday, and find her almost well. She says that she has not been so well for years as she now finds herself. Her countenance is indeed that of health. In swallowing, she takes five or six draughts in rapid succession; but when she swallows leisurely a single mouthful, as I made her do to-day, she does it without coughing. Some weeks after this Report, Fanny Murray placed herself in the way of the surgeon's round, and gave me her thanks in a very eloquent manner, to the amusement of the pupils; and next day she took an unceremonious leave of the hospital."

We learn from Dr. Watson's 'Lectures on the Practice of Physic,' that this was not the only occasion on which Sir Charles Bell applied the solution of the nitrate successfully. A man under Dr. Watson's care, in the Middlesex Hospital, had acute laryngeal disease, for which the operation of laryngotomy was successfully performed by Sir Charles. About a week after the opening in the larynx had closed up, the patient began to wheeze, speak in a hoarse voice, and in a day or two had a paroxysm of extreme dyspnœa. Dr. Watson got Sir Charles to examine the interior of the throat, and they agreed that it would be proper to "swab" the epiglottis and upper part of the air-passages with a strong solution of lunar caustic. Bell applied the sponge with little difficulty, and the next day the breathing was improved, and the hoarseness almost gone. (Lond. Med. Gaz. vol. xxviii, p. 777, Aug. 1841; also Lectures, vol. i, p. 809, first ed., 1843.) Dr. Watson explains, that the naval term, "swabbing," originated with the late Mr. Vance, who was for many years a surgeon in the navy, and who used the application a good deal in his practice. We subjoin Dr. Watson's own words:

"It is said that a little practice will enable a person to pass his finger into a patient's throat, and to familiarize his sense of touch with the ordinary condition of the upper part of the respiratory apparatus, so as to be able to detect swelling, or irregularity, or thickening about the chink of the glottis. And great advantage is said to have been obtained from applying remedies directly to the diseased or irritable part. This practice was much followed by the late Mr. Vance, who had been for many years a naval surgeon, and he called it, in naval phrase, *swabbing* the affected organ. A small piece of sponge, secured with a string, or fastened to the end of the finger of a glove, is dipped in a strong solution of nitrate of silver, and then carried down into the throat, as far as the spasmodic state of the muscles which the attempt induces will permit, and pressed downwards against the superior surface of the larynx. I believe other stimulating applications are sometimes employed in the place of the nitrate of silver. Now, of this method of cure I do not know much, except by report. I have heard that many cases of chronic hoarseness and cough have speedily been cured by it." (Loc. cit., p. 810.)

We repeat, that these cases are not quoted to disparage Dr. Green's researches, but rather as confirmatory of them. The whole subject is undoubtedly his own, by the special attention he has devoted to it. We will now subjoin his method of applying the nitrate. The salt he uses is not the fused, but the crystallized.

"*Method of applying the solution.* In the treatment of laryngeal disease by the direct application of the nitrate of silver to the diseased surface, I have employed ordinarily a solution of this substance, of the strength of from two to four scruples of the nitrate to an ounce of distilled water. When, however, there are found extensive ulcerations of the epiglottis, or about the opening of the larynx—ulcerations which it is desirable to arrest at once—I have not hesitated to apply directly, to the diseased parts, a solution of double the strength of the last named. But one or two applications only of a medicine of this power should be made at one time; ordinarily, however extensive the lesions may be, it will not be necessary to employ a solution of greater strength than one composed of four scruples of the salt to an ounce of water. On the other hand, it has been found, that one of less strength than of from forty to fifty grains of the nitrate to an ounce of fluid will have but little effect upon a diseased mucous surface, where ulcerations exist

"In cases in which it becomes necessary to cauterize the interior of the laryngeal cavity, the aperture of the glottis should not be passed at once; the part should be *educated*, by applying the solution daily, for several days, to the faucial and pharyngeal region, to the epiglottis, and about the opening of the glottis. Proceeding in this manner, that exquisite sensibility whlch belongs to the lips of the glottis, is in a good degree overcome, and the instrument may then be passed into the larynx, without producing half the amount of that irritation which its introduction below the epiglottis would have awakened at first.

"The instrument which I have always employed for making direct medicinal applications into the cavity of the larynx is one composed of whalebone, about ten inches in length (with or without the handle, as represented in the plate), curved at one end, to which is securely attached a small round piece of fine sponge. [The wood-cut in the margin (p. 500) is an exact fac-simile from Dr. Green's plate, and is the full size of the instrnment.]

"The extent to which the rod is to be bent must be varied according to circumstances, for the opening of the glottis is situated much deeper in some throats than in others; but the curve which I have found suited to the greatest number of cases, is one which will form the arc of one quarter of a circle whose diameter is four inches. (See Woodcut, i.)

"The instrument being prepared, and the patient's mouth opened wide and his tongue depressed, the sponge is dipped into the solution to be applied, and, being carried over the top of the epiglottis, and on the laryngeal face of this cartilage, is suddenly pressed downwards and forwards, through the aperture of the glottis, into the laryngeal cavity. This operation is followed by a momentary spasm of the glottis, by which the fluid is discharged from the sponge, and is brought into immediate contact with the diseased surface.

"Every physician who has been present when this operation has been performed (and a large number have witnessed it from time to time) has manifested much surprise on observing how little irritation has been produced by the introduction of the sponge. If the patient, on opening his mouth, take a full inspiration, and then be directed to breathe gently out, at the moment in which the sponge is introduced, the irritation caused by the application will be much less than when this caution is not observed. The fact, indeed, has been fully established by repeated experiments, that the introduction into the larynx of a sponge, saturated with a solution of the crystals of nitrate of silver, of the strength of forty, fifty, or even sixty grains of the salt to the ounce of water, does not produce, ordinarily, as much disturbance as is caused by the accidental imbibition into this cavity of a few drops of tea, or even of pure water!

"In the topical treatment of the follicular disease, it will be found that all larynges cannot be entered with the same facility. Indeed, in some instances, where œdema of the epiglottis and of the arytenoid cartilages has existed, I have found it very difficult in making the first attempt to pass the sponge of the probang through the aperture of the glottis." (pp. 199-202.)

"Nothing will so speedily enlarge the opening of the glottis, when it has been contracted by œdema, as a few applications of the nitrate to the lips of the glottis and the laryngeal face of the epiglottis." (pp. 204-5.)

The use of the solution should be continued for some time: this is practically of great importance, as a relapse will otherwise soon take place. It should be applied at first every other day (and even oftener) for two or three weeks, and then twice a week until the surface assumes a healthy appearance. If the tubercles on the pharynx appear angry or confluent, the solid nitrate may be applied.

Dr. Green justly observes that the muciparous glands of the posterior nares are not unfrequently involved, and an unhealthy mucus is almost incessantly dropping down into the throat. The argentine solution may be applied by a small rod of whalebone, bent at the end into a right angle instead of into a curve.

There is one particular which Dr. Green has omitted to state in the above quotation, and that is, that a spatula must be made expressly for the purpose: a spoon or an ordinary spatula will not serve. It should have a broad blade, 8 inches long and $1\frac{1}{4}$ broad, $4\frac{1}{2}$ inches should be curved at a right angle to the remaining portion, which is to be fixed into a handle 5 inches in length. The blade should be electrotyped with silver, as when made of naked steel its formidable appearance at first sight alarms the patient. Fixed in a handle perpendicular to the curve, the latter depresses the tongue while the hand of the operator is situate below the chin of the patient, and leaves ample room for the application of the solution. The point of the spatula should be carried well backward, so as to touch the root of the tongue, and thus enable the operator not only to depress the tongue, but also to drag it forward. It is by this means that the epiglottis can be brought into view in a very large majority of cases.

Concurrently with this topical application, other local means are necessary. Thus, when the uvula is elongated, partial or total excision is necessary. For this purpose Dr Green uses curved scissors and a pair of long and slender forceps having finely serrated blades. In hypertrophy of the tonsils, Dr. Green urges that excision is the only effectual means, if fibrinous deposit have already taken place. Even homœopathy, Dr. Green observes, seems powerless in these cases. The operation itself is almost painless.

"Hypertrophy and induration of the tonsils occur frequently in young persons and children, independently of follicular disease of the throat. In some instances, the affection appears to be congenital, or is hereditary; in others, it is the result of repeated attacks of chronic inflammation of the tonsillary glands. When the hypertrophy is accompanied by induration, whether this condition coexists with follicular disease, or is the effect of chronic tonsillitis, excision of the enlarged gland is almost the only method of treatment by which permanent and effectual relief can be obtained. This fact ought to be better understood by the profession than it seems to be, for the practice of painting these morbid growths with the tincture of iodine, or of cauterizing them with the solid nitrate, is still continued, and patients are daily being subjected to this annoying and useless practice, often month after month, with the apparent expectation on the part of their attendants that enlarged and indurated tonsils may be discussed by these applications!" (pp. 210-1.)

Dr. Green does not confine the use of the nitrate of silver to the treatment of chronic cases: he applied it to the fauces, pharynx, and larynx, of the strength of 45 grains to the ounce, in a case of acute laryngitis, in which suffocation was imminent. For a few minutes, the difficulty of breathing and the cough were increased by the application; a large amount of viscid ropy mucus was discharged; and along with it a small quantity of blood. In the course of half an hour after the application, the symptoms had improved. The laryngeal cough subsided, the respiration became less laborious, and the patient soon after obtained some sleep. In short, from this hour Dr. Green had no farther trouble from the case. Occasional doses of expectorant medicines were administered during the day. On the following evening, a slight increase of the cough and irritation came on, but these soon subsided, and were followed by a quiet night's rest.

In a case of ulceration of the epiglottis and opening of the larynx in a child, consequent on swallowing sulphuric acid, the remedy was used with the greatest success.

The application of the solution to the bronchi is also advocated and practised by Dr. Green.

"As stated in a foregoing chapter, follicular laryngeal disease is often found coexisting with chronic inflammation of the lining bronchial membrane. The presence of this complication does not contraindicate the topical employment of the nitrate of silver. On the other hand, in making the applications into the laryngeal cavity, a still more free use of the solution should be employed, in order that some part of the fluid may find its way into the bronchial divisions. Among the patients who have come under my care during the last eighteen months, are a number of intelligent physicians, who have been sufferers from laryngeal and bronchial disease. Several of them have informed me repeatedly that, after having a free application of the solution into the larynx, they have felt distinctly the fluid extending down the bronchial tubes. Often, in these cases, no taste of the medicine would be observed until matter, by coughing, was expectorated from the air-passages, when the peculiar flavour of the nitrate of silver—a most acrid bitter—would be perceived; and this would continue to be observed whenever the individual expectorated for many hours after the operation." (pp. 225-6.)

While advocating the argentine solution as an effective topical application, Dr. Green does not appear to have neglected counter-irritants to the spine and throat, and local bleedings. Blisters, leeches, &c., are amongst the remedies of this kind.

We have already referred to the utility and safety of the argentine solution, as a topical application in those cases in which laryngeal disease complicates phthisis pulmonalis. From the experience of twenty-five such cases treated by Dr. Green during the year 1845, he is enabled to speak in strong terms of its value in mitigating the sufferings and prolonging the lives of phthisical patients. The subjoined is a remarkable example of the value of the treatment: it is the case of a gentleman who went from Connecticut to New York:

"Some months before coming to the city he lost his voice entirely; but he was hoarse, and had had a cough, with laryngeal inflammation, several years before this occurrence. He had also had several attacks of hemoptysis; was emaciated, and so feeble when he arrived, that he was not able to walk the distance of a block without aid. He was suffering from a most severe paroxysmal cough; had great dyspnœa, and a free expectoration of purulent matter. The throat was studded with granulations or enlarged follicles, and it was with much difficulty that he could articulate above a whisper. A dullness on percussion over the right lung, with an absence of the respiratory murmur, and pain and stricture about the chest, with the above rational symptoms, marked the case as one of confirmed phthisis; and such it was admitted to be, after a critical exploration of the chest, by several experienced medical friends, among whom was one of the physicians of the New York Hospital, who is esteemed, and justly so, as a most accomplished auscultator.

"Applications of a concentrated solution of the nitrate were made to the throat and into the larynx of this patient for about two weeks. At the end of this time, his cough and dyspnœa were so much relieved, and his strength increased, that from not being able to walk any distance without aid, as was the case when he first arrived, he on the twelfth day of treatment went on foot down to the boat in which he came to the city and back again, a distance of more than three miles, without suffering any inconvenience. After remaining several weeks under treatment, he returned to Connecticut with augmented strength, and with his cough greatly relieved. Nearly three months after he left the city his sister called on me in New York, and informed me that her brother had been able after his return to superintend his affairs, which had not been the case for nearly two years before; and in this favorable condition he remained when last heard from, which was more than a year after he left New York." (pp. 231-3.)

*General treatment of follicular disease.* On looking over the details of the cases treated by Dr. Green, we find that the iodide of potassium holds the first rank. This is conjoined with the sesquioxide of iron, and various sedatives and expectorants, to alleviate the cough. Belladonna, conium, hyoscyamus, and the salts of morphia are among the former class; of the latter are the sanguinaria canadensis, or blood-root, hydrochlorate of ammonia, and the oil of Gualtherica. The sanguinaria is described by Dr. Green as a stimulating expectorant and slightly narcotic; and, when combined with opiates, a valuable remedy in allaying the cough and irritation, in some forms of follicular inflammation, complicated with bronchial or pulmonic disease. The tincture is made by macerating four ounces of the root in two pints of alcohol. From fifteen to twenty minims may be given thrice a day, with five or seven minims of laudanum, and a sixth of a minim of ol. gualthericæ.

We observe that Dr. Green prescribes *one grain* of hydrochlorate of ammonia thrice a day, in combination with squill, digitalis, and opium. We are of opinion that the salt might just as well have been left out. The Germans ordinarily give not less than ten grains for a dose, and although we think this dose unnecessarily large, not less than from three to five grains should be taken.

Mercury in full, but generally in alterative, doses is recommended by Dr. Green. In those cases, he observes, where the general glandular system is involved in the morbid action, or even when some forms of pulmonic lesions coexist with the follicular disease, he has found the bichloride of mercury the most efficient, and, altogether, the best preparation of this mineral.

Exposure to a malarious atmosphere is recommended by Dr. Green in cases of phthisis pulmonalis, and he refers to our review of M. Baudin's publication, as well as to his own experience and that of others, for confirmation of his opinions as to the therapeutic efficacy of a malarious climate. He states no facts, however, that would lead the reader to suppose it could be of benefit in cases of *follicular* disease, nor does he recommend a residence in a marshy district for their cure.

Plates exhibiting the pathological anatomy of follicular disease, and the appearance of the throat in various stages and forms of the affection are appended. There is also a drawing of the instruments employed by the author for making medicinal applications into the laryngeal cavity, for excision of the enlarged tonsils, and for truncating the elongated uvula.

Having thus given an ample analysis of Dr. Green's work, it remains for us to propound briefly a critical estimate of its value. We think that the author has not only made a most valuable addition to practical or empirical medicine, but that the results of his method of treatment will lead to important changes in the prophylaxis and cure of pulmonary phthisis. It would appear from various testifying documents, which the author has collected together in an Appendix, that his statements as to the practicability and safety of topical medication in laryngeal disease was met by some of his countrymen by a sneering incredulity. There can be no doubt, however, that this part of the question is set entirely at rest; nor does the previous publication of the methods used by Bell, Vance, and Trousseau and Belloc, detract at all from the merit due to Dr. Green, for his persevering and successful attempts to render the treatment of some forms of pulmonary disease more effectual and certain. We have adopted this method of treatment recommended by him, and can corroborate his statements as to its great value. Cases of pulmonary affection have in our hands been brought to a satisfactory termination, which we are quite sure, under the treatment ordinarily adopted, would have terminated fatally: and we remember individuals, whose cases terminated fatally, who (we feel equally certain) need not have died, at least of that disease which cut them off. This much is due to Dr. Green.

With regard to the influence of Dr. Green's views, on the prophylaxis and cure of pulmonary phthisis and chronic bronchitis, we would observe that their merit, in this respect, is that of being suggestive. It is of importance to know more precisely what agents determine the deposit of tubercular matter in the lungs; or when deposited, what agents call the latent morbific deposit into activity. The lungs and thorax may be considered as analogous to certain hollow viscera, as the bladder, rectum, uterus, &c. We know that in these there is a point of the mucous membrane at the entrance into the cavity which is endowed with special sensitive power, and the stimulation of which excites expulsive efforts. The laryngo-pharyngeal membrane is endowed with this peculiar sensibility, and as covering the inlet into the stomach and lungs is subservient to the expulsive efforts of retching, vomiting, and coughing; and these efforts must be considered as analogous to tenesmus and dysuria in dysentery and calculus vesicæ.

We find, however, that it is not the muscular apparatus only of these hollow organs that is implicated; the mucous membrane covering their interior is also affected when the sensitive portion of that tissue is irritated, and concurrently with hypertrophy of the muscular fibre there is increased secretion from the mucous membrane, presenting all the different grades of morbid change from simple mucus to a discharge having a purulent character. It becomes then of importance to investigate more accurately than has hitherto been done, the pathological relations of these sensitive inlets to the mucous surfaces with which they are in such intimate connexion, and more especially the pathological relations of the pharyngo-laryngeal membrane to the pulmonary structures, including both the external and internal. If upon inquiry it be found, that a morbid condition of the muciparous glands of that membrane, or of the membrane itself, be a frequent determining or exciting cause of tubercular deposit and of those various structural changes in the pulmonary mucous membrane, generalised under the term *chronic bronchitis*, the physiology and pathology of those glands well demand a close investigation. We are inclined to think that the condition of the abdominal viscera will be found to have an important influence on them. There is a close relation between the two extremities of the alimentary canal: this is shown in those cases of phthisis pulmonalis complicated with fistula in ano, and of which we do not remember an example that was not complicated with pharyngo-laryngeal disorganization. In cases of phthisis complicated with a gouty diathesis, and also with hepatic disease, we have frequently observed a similar complication; and our late experience leads us to think, that in such cases the symptoms might be much ameliorated, life much prolonged, and perhaps the more fatal pulmonary affection prevented by a timely attention to the state of the respiratory inlets.

The predisposing causes of pulmonary disease may also be eventually better elucidated than hitherto. The practice of smoking tobacco, especially in the form of cigars, cannot fail to set up a morbid action in the pharynx and larynx, and so induce insidious chronic inflammation of the pulmonary membrane; and we can fully corroborate Dr. Green's statements on this head. The low degree of sensibility in the fauces generally, renders it necessary in all cases not to trust to the declaration of the smoker, that he feels no pain or uneasiness in the throat. The practitioner must inspect it, and in many examples he will find follicular disease in its first and second stages, although no complaint has been made by the patient. Nor should the quantity smoked be a guide to his judgment in determining the amount of mischief, as there is the widest difference between individuals in the amount of tolerance of the "weed;" a single cigar in one individual will produce as bad effects as half a dozen in another. We cannot, however, here enter more fully into the suggestions we have thrown out. The scientific reader will readily see that the etiological and pathological relations of the throat are very numerous, and that it is for him to work out practical results by daily observation.

We ought not to close this article, without suggesting to Dr. Green the propriety of correcting the proof-sheets of any future volume he may publish, for we think it is impossible he can have done that necessary duty on the present occasion. The grossest errors abound, in punctuation, in the spelling of professional terms, and in the latinity of the formulæ. The Latin of medical rescripts is already ridiculously *doggish,* even when correctly written; but what shall we say to such a confusion of genders and cases as in the following:

"℞. Hydrarg. chlo. corrosiv. gr. xii. Solve in aq. distil. q. s. adde Micæ Panis Alba, Saccharis Alba āā q. s. ut fit pilulæ numero ccxl."

Or this:

℞. Tinct. Sanguinaria ℥iss.
Tinct. Opii. ℥ss.
Ol. Gaultheria gtt. x.
Misce; cujus capiat guttas viginti vel triginti, ter die.

Our brethren in the United States are so closely allied to us by community of origin, of manners, and of language, that we have a family jealousy of any aberration from those minutiæ of composition, which render a very attractive subject the more attractive, and the absence of which diminishes the merits of the best book.

## Figures and Tables

**Figure f1:**